# Highly Pathogenic Avian Influenza A(H5N6) in Domestic Cats, South Korea

**DOI:** 10.3201/eid2412.180290

**Published:** 2018-12

**Authors:** KyungHyun Lee, Eun-Kyoung Lee, HyunKyoung Lee, Gyeong-Beom Heo, Yu-Na Lee, Ji-Youl Jung, You-chan Bae, ByungJae So, Youn-Jeong Lee, Eun-Jin Choi

**Affiliations:** Author affiliation: Animal and Plant Quarantine Agency, Gimcheon, South Korea

**Keywords:** highly pathogenic avian influenza, HPAI, cats, pathology, H5N6, viruses, influenza, influenza A, South Korea

## Abstract

In December 2016, highly pathogenic avian influenza (HPAI) infection with systemic pathologic lesions was found in cats in South Korea. Genetic analyses indicated that the feline isolates were similar to HPAI H5N6 viruses isolated in chicken farms nearby. This finding highlights the need for monitoring of domestic mammals during HPAI outbreaks.

Highly pathogenic avian influenza (HPAI) H5N6 has spread across Asia, Europe, and Africa. Since a novel influenza A(H5N6) virus emerged in China in late 2013 (*1*), H5N6 viruses have been subsequently reported in Southeast Asia. In China, HPAI A(H5N6) virus caused the earliest reported human infection in 2014 and became one of the dominant subtypes in poultry farms and live poultry markets (*2*). These viruses caused a potential threat to other mammals, including pigs and cats (*3*,*4*). We report H5N6 virus infection in cats during 2016–17 HPAI outbreaks in domestic poultry in South Korea (*5*).

## The Study

**Figure 1:**
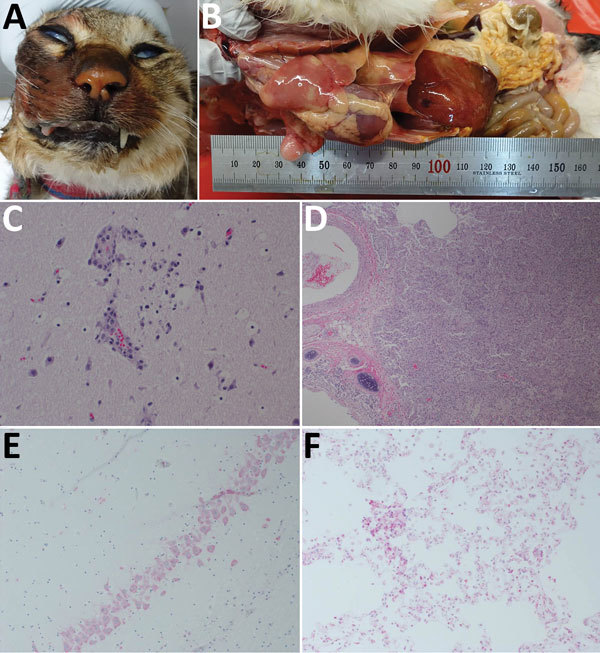
Gross, microscopic, and immunohistochemical (IHC) findings in 3 cats with highly pathogenic avian influenza A(H5N6) virus infection, South Korea. A) Bloody nasal discharge. B) Severe congestion and edema in the lungs and white-colored foci in the liver. C) Gliosis in the brain. Hematoxylin and eosin stain; original magnification ×100. D) Interstitial pneumonia with degenerated pneumocytes. Hematoxylin and eosin; original magnification ×40. E) Influenza viral antigens in neurons. IHC testing; original magnification ×100. F) Influenza viral antigens in alveolar macrophages. IHC testing; original magnification ×100.

The 2016–17 winter season saw epidemics of HPAI A(H5N6) in domestic poultry and wild birds in South Korea (*5*). At the end of December 2016, three carcasses of cats were submitted from areas near H5N6 virus–infected chicken farms in Pocheon. The cats had shown sudden clinical signs of salivation, lethargy, convulsion, and bloody discharge around the mouth and jaws and died within 4 days after illness onset despite antimicrobial drug treatment. After necropsy, we processed representative tissues for histopathology and immunohistochemistry (Technical Appendix). The necropsy findings included bloody nasal discharge (Figure 1, panel A), severe pulmonary congestion and edema, and white-colored foci in the liver (Figure 1, panel B). The lungs were red and yellow in color and incompletely collapsed and had accumulated a small amount of frothy fluid. The spleen was enlarged 2-fold. The pancreas showed spotty hemorrhage and white pinpoint foci.

Histopathologic examination revealed severe lesions in brain, lungs, and liver in the examined cats. We observed necrosis and loss of neurons, lymphocytic perivascular cuffing, and gliosis (Figure 1, panel C) in the cerebellum and cerebrum, and especially severe necrosis in the hippocampus. The lungs showed marked congestion, edema, hemorrhage, and severe interstitial pneumonia (Figure 1, panel D), and thrombus in the alveolar capillaries. The liver showed severe necrotic foci and hepatitis. We observed influenza viral antigen in neurons (Figure 1, panel E), glial cells, and alveolar macrophages (Figure 1, panel F). Table 1 describes histopathologic lesions and Table 2 immunohistochemical reactivity.

**Table 1:** Pathologic lesions in various tissues of 3 cats diagnosed with highly pathogenic avian influenza, South Korea

Tissue	Lesions	Cat 1	Cat 2	Cat 3
Brain	Neuronal necrosis	Severe	Severe	Mild
	Meningoencephalitis	Moderate	Moderate	Minimal
	Gliosis	Moderate	Moderate	Minimal
Trachea	Lymphocytic tracheitis	Minimal	Minimal	Minimal
Lung	Interstitial pneumonia	Severe	Severe	Severe
	Pneumocytic necrosis	Severe	Moderate	Severe
Heart	Myocytic necrosis	Minimal	Minimal	Minimal
	Lymphocytic myocarditis	Minimal	Minimal	Minimal
Spleen	Lymphocytic necrosis	Minimal	Minimal	Minimal
	Lymphocytic depletion	Mild	Minimal	Minimal
Pancreas	Acinar epithelial necrosis	Minimal	None	None
Intestine	Enterocytic necrosis	Minimal	None	None
	Enteritis	Minimal	None	None
Liver	Hepatic necrosis	Severe	Severe	Severe
Kidney	Tubular necrosis	None	None	None
	Nephritis	None	None	None

**Table 2:** Reactivity to influenza viral nucleoprotein in various tissues of 3 cats with highly pathogenic avian influenza, South Korea

Tissue	Cells	Reactivity		
		Cat 1	Cat 2	Cat 3
Brain	Neurons	Numerous	Numerous	Moderate
	Glial cells	Moderate	Numerous	Minimal
	Ependymal cells	Numerous	Numerous	Minimal
Trachea	Epithelial cells	Minimal	Minimal	Minimal
Lung	Macrophages	Numerous	Numerous	Numerous
	Vascular endothelial cells	Numerous	Numerous	Numerous
Heart	Myocytes	Minimal	Minimal	Minimal
	Epicardial cells	Minimal	Minimal	Minimal
Spleen	Ellipsoid capillary endothelium	Minimal	Minimal	Minimal
	Macrophages and necrotic debris	Moderate	Minimal	Minimal
Pancreas	Acinar epithelium	Minimal	None ^–^	None
Intestine	Crypt epithelium	Minimal	None–	None
	Mesenteric ganglial neurons	Minimal	None	None
Liver	Kupffer cells and necrotic debris	Numerous	Numerous	Numerous
Kidney	Tubule epithelium	Minimal	Minimal	Unknown
	Glomeruli	Minimal	None	None

We recognized H5N6 virus infection in a domestic male cat (cat 1) and juvenile outdoor cats (cats 2 and 3). We observed necrotic lesions and influenza viral antigens in multiple visceral organs, suggesting that the virus caused systemic infection. It seems likely that the neurotropism of H5N6 virus was a key factor contributing to the sudden death of these cats. The results of this study are consistent with those of other studies of HPAI pathogenicity in experimentally infected dogs (*6*,*7*).

The histopathologic findings and the localization of H5N6 virus antigen to the lungs and liver, but not to the brain, in cats have been reported (*8*). In this case, we observed meningoencephalitis. Moreover, the 3 cats showed neurologic symptoms such as salivation and convulsion, which may be related to necrosis and loss of neurons. The severity of the lesions was consistent with the number of cells that reacted with influenza viral antigen. A few studies reported that H9 and H10 influenza viruses were nephrotropic in chickens with low pathogenicity (*9*,*10*) and that HPAI H5 virus causes acute renal lesions in mammals and primates, including humans (*11*). The results of our study suggest that the HPAI H5N6 virus affects cats differently than do other HPAI viruses; therefore, further studies are needed to experimentally infect cats with other HPAI H5 subtypes, including the isolate from this study, for complete clarification.

Previous studies have shown that avian viruses preferentially recognize α-2,3 linkage (SA α 2,3Gal) and bind to type II alveolar cells, which are abundant in the lower respiratory tract of mammals (*12*,*13*). These findings are consistent with our observations of severe pneumonia with lung edema in the infected cats.

**Figure 2:**
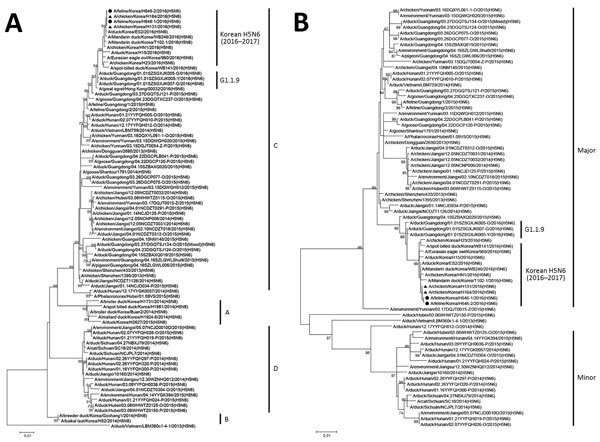
Maximum-likelihood phylogenetic tree of the hemagglutinin (A) and neuraminidase (B) gene segments for highly pathogenic avian influenza A(H5N6) viruses from cats, South Korea, and comparison viruses. Black circles indicate isolates from cats and triangles indicate chicken isolates from this study. Virus sequences from the GISAID EpiFlu database (http://platform.gisaid.org) and GenBank were used for each phylogenetic comparison. The genetic subclades are annotated to the right of the tree. The genetic clusters major, minor, and G1.1.9 were designated according to the criteria of Bi et al. (*2*). The number at each branch indicates a bootstrap value. Scale bars indicate nucleotide substitutions per site.

RNA samples extracted from organs of the cats were positive for influenza H5 and N6 subtypes by reverse transcription PCR. We selected 2 HPAI H5N6 viruses: A/feline/Korea/H646-1/2016(H5N6) from the domestic male cat and A/feline/Korea/H646-2/2016(H5N6) from 1 juvenile outdoor cat. We performed virus isolation, sequencing, and phylogenetic analysis as described (online Technical Appendix). Phylogenetic analyses showed that the H5 genes of the cat isolates belonged to clade 2.3.4.4.C and were very closely related to H5N6 viruses detected in poultry in areas within a radius of 1 km of the cats’ location (Figure 2, panel A). In a previous study, clade 2.3.4.4.C H5N6 viruses from the 2016–17 epidemic in South Korea were divided into 5 distinct genogroups (C-1 to C-5) (*5*). The feline isolates showed high similarity with H5N6 viruses in genogroup C-4, which was detected in domestic poultry nearby during 2016–17 HPAI A(H5N6) outbreaks (Figure 2; Technical Appendix Figure).

Epidemiologic studies show that the cats might be infected by feeding on or by contact with infected wild birds, although the virus was not isolated from wild birds around this area. The affected domestic cat lived in a house near a small stream where migratory birds were observed and a wide main road. Across the main road, H5N6 virus–affected chicken farms were located within 1 km. In previous reports, cats and tigers were naturally infected by feeding on infected bird carcasses (*8*,*14*). In China, H5N6 virus infection in cats has been reported in regions such as Suchuan and Guangdong Provinces (*3*).

We compared each gene of the feline and chicken H5N6 isolates (online Technical Appendix Table). The hemagglutinin (HA) genes of the viruses contained multiple basic amino acid residues at the HA cleavage site (PLRERRRKR). The amino acid residues on the receptor binding sites of the HA gene of H5N6 viruses were Q226 and G228 (H3 numbering), indicating an avian-like (α2,3-SA) receptor-binding preference. T160A mutation in the HA gene suggested a possible increased viral affinity for human-like (α2,6-SA) receptor binding, shown in feline isolates. The neuraminidase genes of feline isolates also had 11 aa deletions at positions 59–69, which were often observed in avian influenza virus lineages adapted to poultry and may increase the virulence to mammals (*2*). We did not observe amino acid substitution at position E627K of the polybasic 2 gene in the feline isolates.

## Conclusions

Our results demonstrate that cats can be directly infected by HPAI H5N6 virus. Cats are companion animals and may act as a vector for influenza transmission to humans. Despite the low probability of H5N6 virus infection in companion animals, avian influenza surveillance will be needed for mammals, including cats, during H5N6 outbreaks.

Technical Appendix Additional information about HPAI A(H5N6) virus in domestic cats, South Korea.
