# Supplementary material for: Highly Pathogenic Avian Influenza A(H5N6) in Domestic Cats, South Korea
Source: Emerg Infect Dis. 2018 Dec;24(12):2343–7. doi: 10.3201/eid2412.180290 (PMC6256404; doi:10.3201/eid2412.180290)
Supplement: Technical Appendix — Additional information about HPAI A(H5N6) virus in domestic cats, South Korea. [file 18-0290-Techapp-s1.pdf]

Article DOI: https://doi.org/10.3201/eid2412.180290

Highly Pathogenic Avian Influenza A(H5N6) in Domestic Cats, South Korea

Technical Appendix

Materials and Methods

Virus Isolation and Sequencing

Viral RNA extracted from organs of dead cats using a Patho Gene-spin DNA/RNA extraction kit (iNtRON Biotechnology, Sungnam, Korea) according to the manufacturer's instructions were positive for H5 and N6 subtype by RT-PCR (*1*,*2*). For virus isolation, the specimens were inoculated into embryonated specific pathogen-free chicken eggs. After incubation at 37°C, allantoic fluid from inoculated eggs was harvested and tested using the hemagglutination assay. All 8 RNA genomic segments were amplified using segment specific primers and directly sequenced (*3*). The nucleotide sequences of the 8 RNA genomic segments of 2 H5N6 isolates, A/feline/Korea/H646–1/2016(H5N6) and A/feline/Korea/H646–2/2016(H5N6), were deposited to GISAID (accession nos. EPI1123314–21 and EPI1123332–39). In addition, the nucleotide sequences of 2 H5N6 viruses [A/chicken/H131/2016(H5N6) and A/chicken/H164/2016(H5N6)] isolated from chicken farms in Pocheon-gun of Gyeonggi-do province in December 2016, respectively, were deposited to GISAID (EPI1123348–55 and EPI1123340–47).

Phylogenetic Analysis

The nucleotide sequences for phylogenetic analysis were downloaded from GISAID (http://www.gisaid.org) or GenBank. All nucleotides optimization and multiple sequence alignment were performed by CLC main workbench software; version 6.8.2 (CLC bio, Aarhus, Denmark). The maximum-likelihood (ML) trees were conducted by MEGA version 6.0 (www.megasoftware.net) using complete coding nucleotide sequences of 8 segments. The nucleotide substitution models (HA, NP, and NA, Hasegawa-Kishino-Yano with Gamma-distribution; PB2, PB1, PA, General Time Reversible with Gamma-distribution; M, Kimura 2-

parameter with Gamma-distribution; NS, Tamura 3-parameter with Gamma-distribution) of each segment for ML trees were computed by MEGA 6 software package (*4*,*5*). The test of phylogeny was estimated by bootstrap analysis with 1000 replication. Phylogenetic trees constructed for the PB2, PB1, PA, HA, NP, NA, M and NS segments are shown in Figure 2 and online Technical Appendix Figure.

Histopathologic and Immunohistochemical Test

Collected tissues (brain, heart, lung, spleen, kidney, liver, pancreas, intestine) were fixed for 24 hours in 10% buffered neutral formalin and processed for paraffin embedding. Paraffin-embedded sections were cut (4 µm), dewaxed, and stained with hematoxylin and eosin. Duplicate sections were immunohistochemically analyzed to determine the distribution of influenza virus antigens in individual tissues. Briefly, sections were reacted with a monoclonal antibody against influenza A virus nucleoprotein (MCA-400; AbD Serotec, Dusseldorf, Germany), followed by a biotinylated goat anti-mouse IgG as secondary antibody and an avidin-biotin complex system (Ventana Medical Systems, Tucson, AZ, USA). The RedMap Kit (Ventana Medical Systems) served as a chromogen substrate. Slides prepared with serial sections of the same tissues were incubated with PBS instead of antibody as a negative control.

References

1. Munch M, Nielsen LP, Handberg KJ, Jørgensen PH. Detection and subtyping (H5 and H7) of avian type A influenza virus by reverse transcription-PCR and PCR-ELISA. Arch Virol. 2001;146:87–97. PubMed http://dx.doi.org/10.1007/s007050170193

2. Fereidouni SR, Starick E, Grund C, Globig A, Mettenleiter TC, Beer M, et al. Rapid molecular subtyping by reverse transcription polymerase chain reaction of the neuraminidase gene of avian influenza A viruses. Vet Microbiol. 2009;135:253–60. PubMed http://dx.doi.org/10.1016/j.vetmic.2008.09.077

3. Hoffmann E, Stech J, Guan Y, Webster RG, Perez DR. Universal primer set for the full-length amplification of all influenza A viruses. Arch Virol. 2001;146:2275–89. PubMed http://dx.doi.org/10.1007/s007050170002

4. Nei M, Kumar S. Molecular evolution and phylogenetics. New York: Oxford University Press; 2000.

5. Tamura K, Stecher G, Peterson D, Filipski A, Kumar S. MEGA6: Molecular Evolutionary Genetics Analysis version 6.0. Mol Biol Evol. 2013;30:2725–9. PubMed http://dx.doi.org/10.1093/molbev/mst197

6. Bi Y, Chen Q, Wang Q, Chen J, Jin T, Wong G, et al. Genesis, evolution and prevalence of H5N6 avian influenza viruses in China. Cell Host Microbe. 2016;20:810–21. PubMed http://dx.doi.org/10.1016/j.chom.2016.10.022

7. Lee EK, Song BM, Lee YN, Heo GB, Bae YC, Joh SJ, et al. Multiple novel H5N6 highly pathogenic avian influenza viruses, South Korea, 2016. Infect Genet Evol. 2017;51:21–3. PubMed http://dx.doi.org/10.1016/j.meegid.2017.03.005

Technical Appendix Table. Comparison of the molecular characteristics of the H5N6 highly pathogenic avian influenza viruses in South Korea*

			Antigenic site of HA			HA cleavage site	NA deletion	M2		NS	NS deletion		PB2		PB1-F2	
Strain	Host	Genogroup	156 (160)†	222 (226)†	224 (228)†	321–330 (333–339)†	59–69	26	31	42	80–84	218–230	627	701	66	Length
A/chicken/Korea/H131/2016 (H5N6)	Poultry	C4	A	Q	G	PLRERRRKR	Yes	L	S	S	Yes	No	E	D	N	90
A/chicken/Korea/H164/2016 (H5N6)	Poultry	C4	A	Q	G	PLRERRRKR	Yes	L	S	S	Yes	No	E	D	N	90
A/feline/Korea/H646–1/2016(H5N6)	Feline	C4	A	Q	G	PLRERRRKR	Yes	L	S	S	Yes	No	E	D	N	90
A/feline/Korea/H646–1/2016(H5N6)	Feline	C4	A	Q	G	PLRERRRKR	Yes	L	S	S	Yes	No	E	D	N	90
Avian influenza viruses	–	–	T	Q	G	–	Yes	L	S/N	S/A	Yes	No/ deletion	E	D	N	–
Human influenza viruses	–	–	A	I/L	S	–	Yes	I	S/N	S	No	No	K	N	S	–

*–, Not determined.
†H3 numbering.

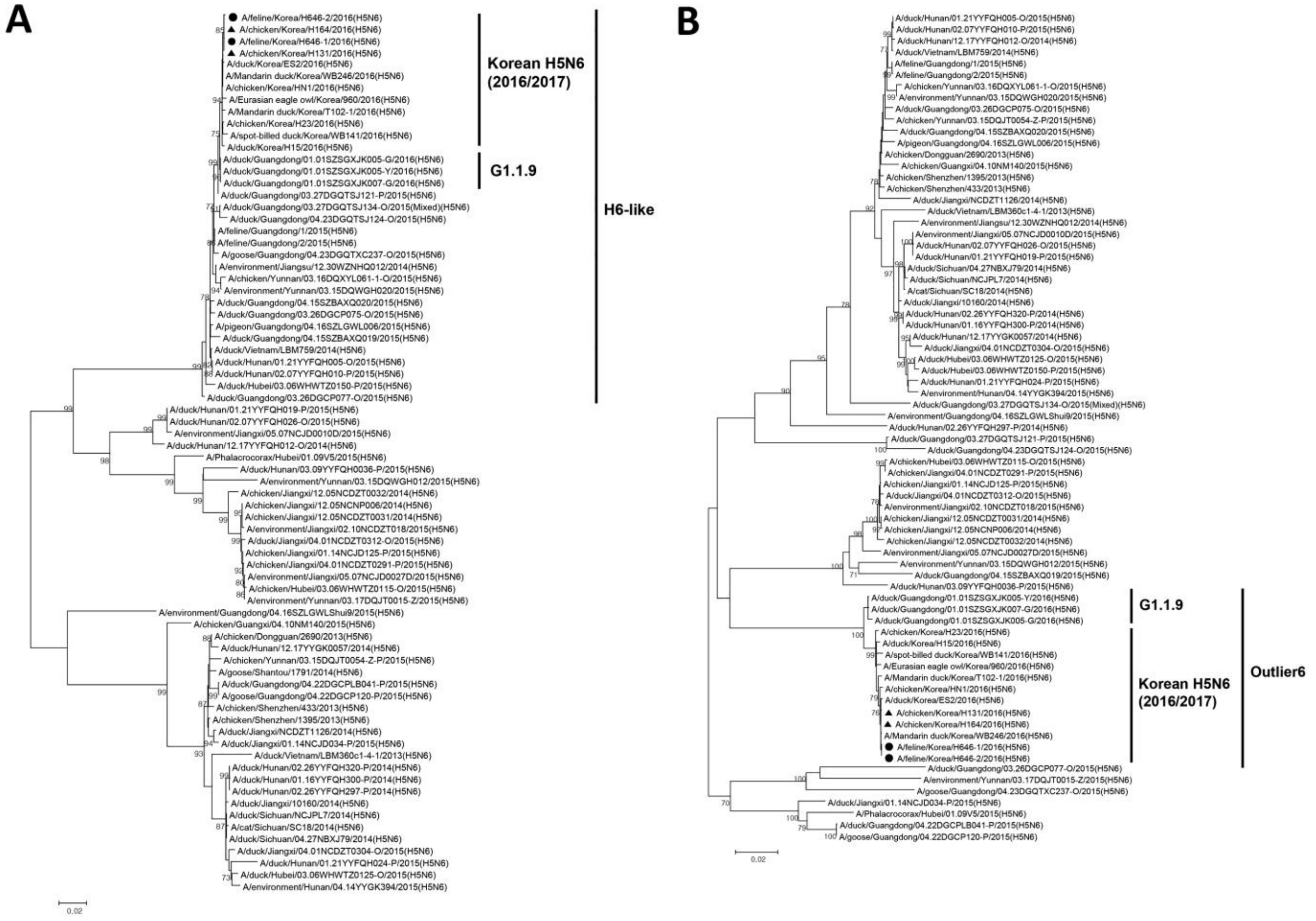
A
Korean H5N6
(2016/2017)
G1.1.9
H6-like
0.02
B
G1.1.9
Korean H5N6
(2016/2017)
Outlier6
0.02

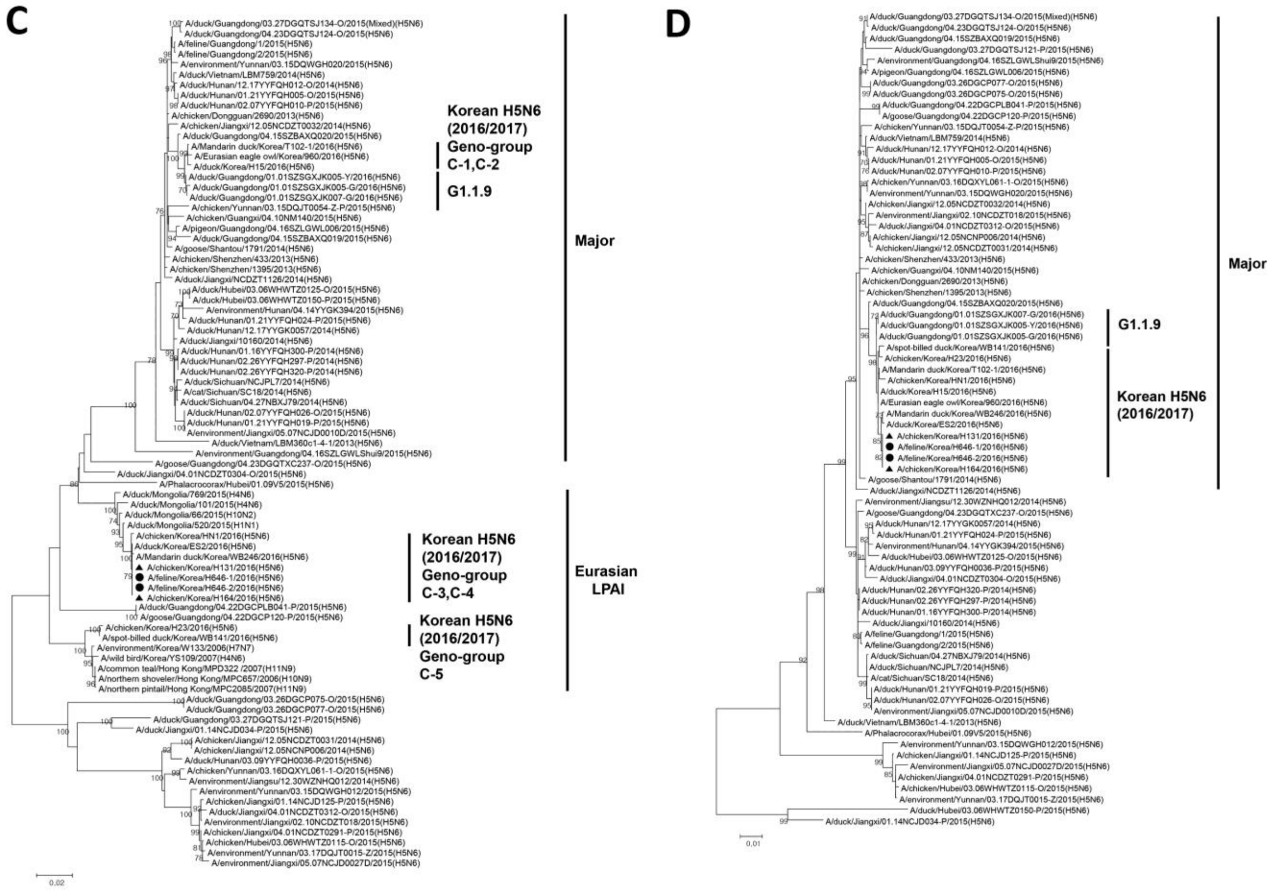
C
Korean H5N6
(2016/2017)
Geno-group
C-1,C-2
G1.1.9
Major
Korean H5N6
(2016/2017)
Geno-group
C-3,C-4
Eurasian
LPAI
Korean H5N6
(2016/2017)
Geno-group
C-5
D
Major
G1.1.9
Korean H5N6
(2016/2017)

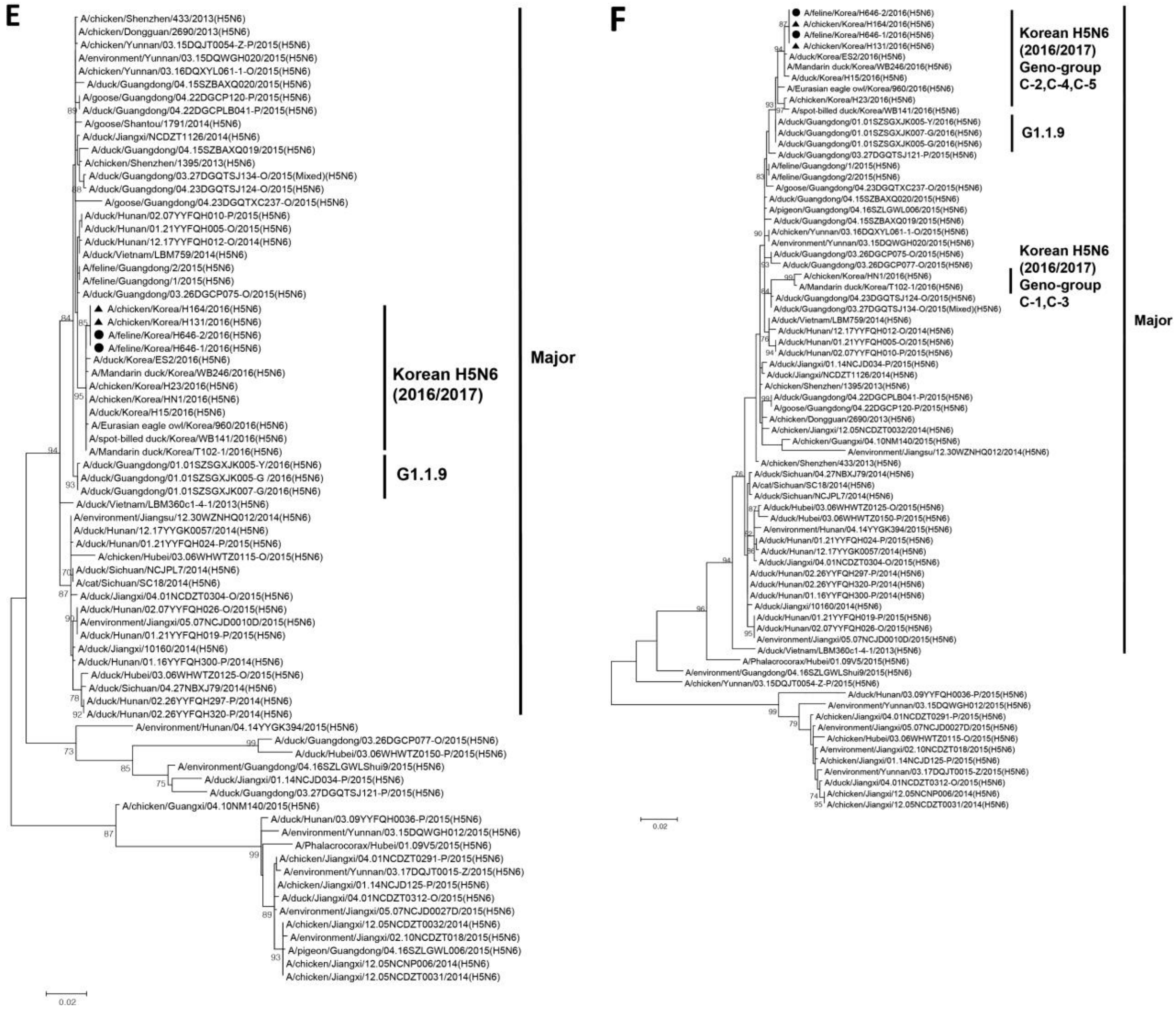

Technical Appendix Figure (following pages)**.** Maximum-likelihood phylogenetic tree for internal gene segments for the feline H5N6 viruses and references**.** A) Polymerase basic-2 (PB2); B) polymerase basic-1 (PB1); C) polymerase acidic (PA); D) nucleoprotein (NP); E) matrix (M); F) nonstructural (NS). The highly pathogenic and low pathogenicity influenza virus sequences from the GISAID EpiFlu database (http://platform.gisaid.org/epi3/frontend#326742) were used for each phylogenetic comparison. The genetic subclades are annotated to the right of the tree. The genetic clusters; Major, Minor and G1.1.9, were designated according to the criteria of Bi et al. (*6*) and the genogroups of Korean H5N6 HPAI viruses (C-1 to C-5) are annotated to the right of the tree of PA and NS (*7*). At each branch, the number indicates a bootstrap value (>70%). Black circles indicate feline isolates in this study and triangles indicate chicken isolates. Scale bar indicates nucleotide substitutions per site.
